# Perspectives on tissue-selective insulin derivatives

**DOI:** 10.1098/rsob.260175

**Published:** 2026-10-14

**Authors:** Maxmilián Hejl, Jiří Jiráček

**Affiliations:** ^1^ Institute of Organic Chemistry and Biochemistry, Czech Academy of Sciences, Prague, Czech Republic; ^2^ Department of Biochemistry, Faculty of Science, Charles University, Prague, Czech Republic

**Keywords:** tissue targeting, diabetes, metabolism, hormone engineering, conjugate

## Abstract

Insulin is a central regulator of metabolic homoeostasis with diverse, tissue-specific actions across multiple organs. Despite major advances in insulin formulations, current therapies rely on systemic delivery and do not reproduce the physiological distribution of endogenous insulin, resulting in non-physiological tissue exposure and associated metabolic limitations. In this perspective, we discuss emerging strategies aimed at achieving tissue-selective insulin action, including molecular engineering of insulin analogues, modulation of insulin receptor isoform specificity and targeted delivery approaches. Attention is given to hepato-preferential, muscle-directed, brain-targeted and adipose-modulating strategies, highlighting their mechanistic basis, therapeutic potential and inherent limitations. While proof of principle has been established for several approaches, challenges remain in balancing tissue selectivity with whole-body metabolic control. Advances in protein design and delivery technologies may ultimately enable the development of next-generation insulin therapies that better mimic physiological insulin action and improve clinical outcomes in metabolic and neurodegenerative diseases.

## 1. Introduction

Tissue- and site-selective therapeutic approaches have emerged as a broadly applicable concept aimed at preferential delivery of drugs, biologics or gene-based interventions to specific tissues, cell types or pathological sites, while limiting exposure of healthy tissues. By concentrating therapeutic activity at the site of disease, such approaches seek to enhance efficacy, reduce systemic side effects and improve the overall therapeutic index. Over the past decade, this paradigm has gained increasing attention across diverse areas of medicine, including oncology, infectious diseases, immunology and metabolic disorders (comprehensively reviewed in [1]), thereby realizing, at least in part, Paul Ehrlich's original ‘magic bullet’ concept of selectively targeting diseased cells while sparing healthy tissues [2].

Multiple strategies have been developed to achieve tissue selectivity ( figure 1). Small molecules, such as monosaccharides, folic acid and urea derivatives, can serve as targeting moieties for tissue- or compartment-specific delivery through specific interactions with receptors such as mannose-6-phosphate receptor [3], GLUT1 [4], ASGP receptor [5], the folate receptor [6] or prostate-specific membrane antigen [7], respectively. Aptamers are another well-studied class of targeting ligands. These single-stranded oligonucleotides represent relatively low-molecular-weight molecules (up to approx. 15 kDa) capable of binding specific proteins and directing conjugated cargos to defined compartments [8].

**Figure 1.**
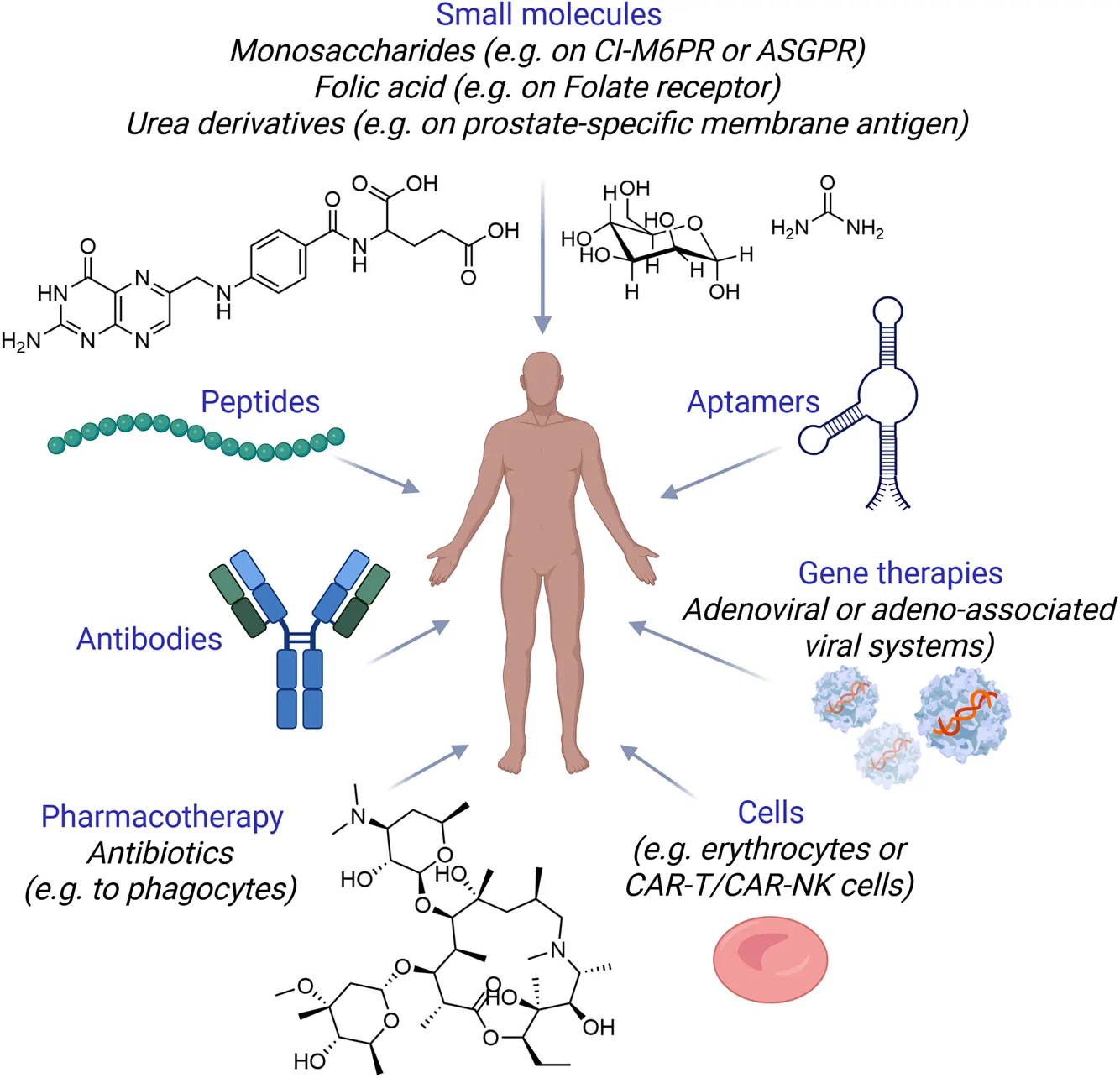
General strategies for achieving tissue selectivity. Examples of molecules or approaches that can serve as targeting moieties for tissue- or compartment-specific delivery via specific interactions. Further details are provided in the text. Created in BioRender.

Owing to the ease of their chemical synthesis and modification, peptides represent an attractive and versatile platform for selective targeting, enabling both the direct conjugation of drugs to targeting peptides (peptide–drug conjugates) and the generation of receptor-specific peptide ligands capable of targeting, for example the central nervous system (CNS) and the blood–brain barrier (BBB) [9].

Antibody-based therapies exploit cell-surface antigens to selectively deliver drugs, such as antibody–drug conjugates (ADCs), to defined cell populations. The remarkable clinical progress of this strategy is underscored by the growing number of approved ADCs and the rapid expansion of the field. As of 2025, 15 ADCs have received Food and Drug Administration (FDA) approval spanning more than 15 cancer indications, while more than 1300 ADC-related clinical trials are ongoing worldwide, according to recent industry reports [10,11].

Gene therapy has similarly adopted tissue-directed principles, using viral vectors (e.g. adenoviral and adeno-associated virus systems) engineered to enhance tissue tropism and/or restrict transgene expression through tissue- or disease-context–specific regulatory elements, thereby enabling preferential activity in tumours, inflamed tissues or metabolically relevant organs [12,13].

In infectious diseases, tissue-directed pharmacotherapy may also arise from intrinsic drug properties, as illustrated by azithromycin, which accumulates intracellularly – particularly in phagocytic cells—and is thereby delivered at high concentrations to sites of infection [14,15].

Cell-based targeting strategies, although still in an early stage of development, represent one of the most promising avenues towards highly selective, biologically active drug delivery systems. Therapeutics may be encapsulated within cells or attached to the cell surface, including the ‘cellular backpack’ concept, which minimizes lysosomal degradation of the payload while preserving cell migratory function [16]. Among cell carriers, red blood cells are the most extensively studied owing to their long lifespan (approx. 120 days in humans) and natural immune tolerance. Red blood cell–based systems are particularly suited for circulatory targeting and sustained drug release and are the most studied cell model. Clinically advanced examples include EryDex®, a red blood cell–encapsulated dexamethasone formulation currently in phase III trials for ataxia telangiectasia, as well as red blood cell–loaded asparaginase for oncological indications [1]. Moreover, senescent red blood cells can be exploited for spleen-specific targeting, enabling antigen delivery and induction of immunological tolerance in autoimmune disease models [17]. In parallel, engineered immune cells, such as chimeric antigen receptor T (CAR-T) cells and CAR-natural killer (CAR-NK) cells, have emerged as highly promising cell-based targeting platforms capable of selective recognition and elimination of diseased cells. These therapies rely on genetic modification of immune cells to express synthetic receptors recognizing tumour-associated antigens independently of major histocompatibility complex presentation, thereby enabling potent and highly specific antitumor responses. CAR-T cell therapies have already demonstrated remarkable clinical efficacy in haematological malignancies, leading to multiple FDA approvals, while CAR-NK cells are being intensively investigated as a potentially safer and more accessible alternative with reduced risk of cytokine release syndrome, lower graft-versus-host reactivity and the potential for ‘off-the-shelf’ allogeneic applications [17–20].

Despite their diversity, tissue-directed therapeutic strategies share common underlying principles [1,20,21]. Effective tissue targeting requires achieving sufficiently high local drug concentrations at the disease site together with molecular, cellular or anatomical specificity that discriminates pathological from healthy tissues. Whether such selectivity can be achieved and sustained following systemic administration is governed by biological barriers and pharmacokinetic factors, including circulation time, tissue distribution and clearance. Tissue preference may arise from intrinsic drug properties, rational drug design, delivery vehicles (e.g. nanoparticles, extracellular vesicles and tissue-engineered scaffolds) or vector- and device-based strategies enabling localized or prolonged exposure. Collectively, these approaches enhance therapeutic efficacy, reduce off target toxicity and improve control over drug kinetics, providing a useful framework for evaluating established and emerging therapeutic modalities, particularly in systemic diseases with tissue-restricted dysregulated signalling.

### 1.1. Insulin: a central hormone with tissue-specific actions

Insulin represents a particularly instructive example of a bioactive compound with broad systemic effects and major therapeutic importance. This polypeptide hormone [22], produced by pancreatic *β*-cells and secreted in response to elevated blood glucose levels, plays a central role in maintaining metabolic homoeostasis by promoting glucose storage in the liver, skeletal muscle and adipose tissue. Beyond its classical metabolic functions, insulin exerts diverse, tissue-specific effects throughout the body, ranging from developmental roles to distinct actions in the CNS [23].

Clinically, insulin remains an essential therapy for millions of patients worldwide [24,25]. For individuals with type 1 diabetes, insulin replacement is life-saving, while a substantial proportion of patients with type 2 diabetes ultimately require exogenous insulin to compensate for progressive insulin resistance. Despite major advances in insulin formulations and delivery technologies, current therapeutic strategies remain fundamentally non-physiological. In contrast to endogenous insulin secretion—where insulin reaches the liver first via the portal circulation—therapeutic insulin is typically administered subcutaneously, resulting in altered tissue distribution and exposure (figure 2).

**Figure 2.**
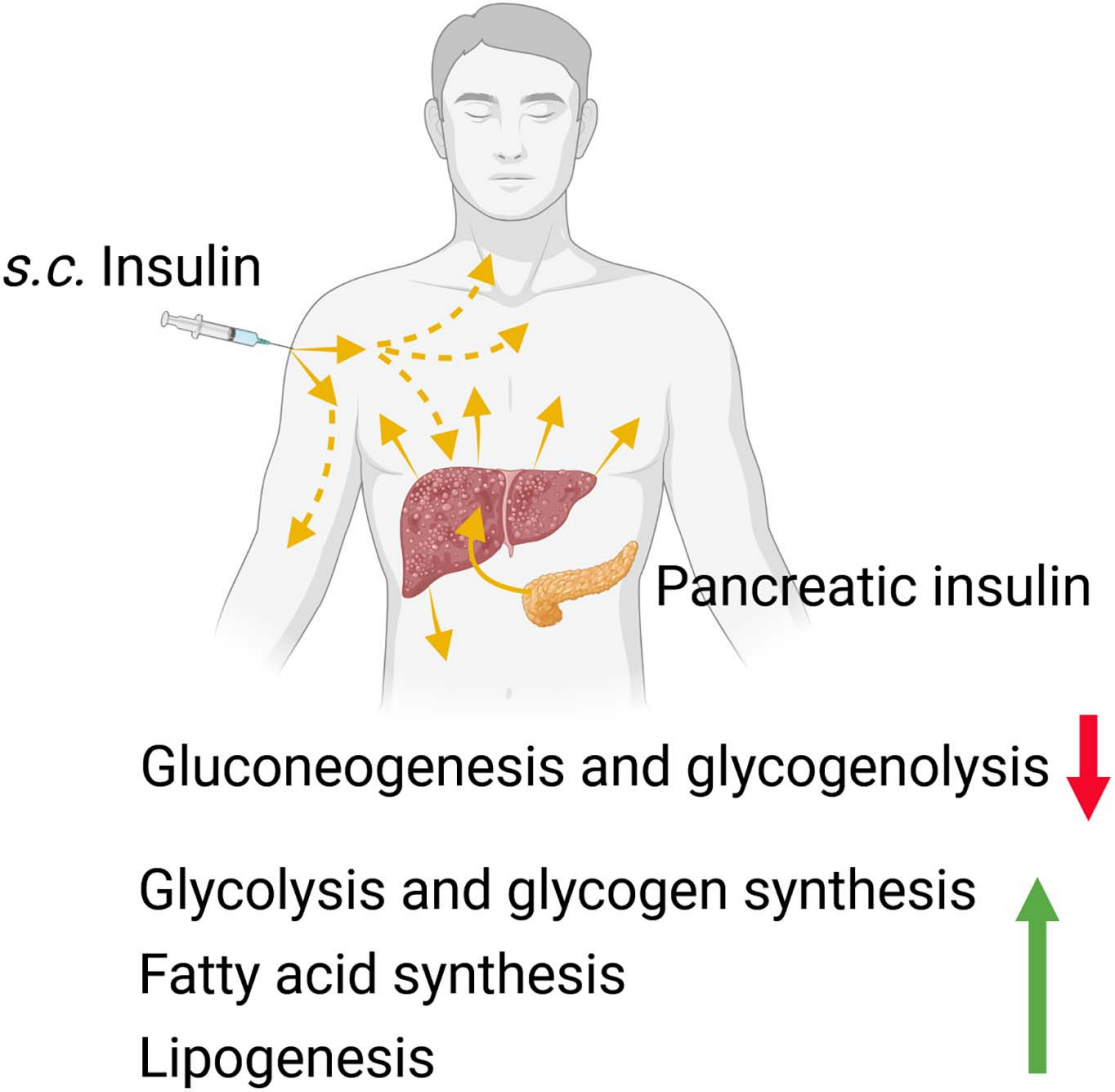
Hepatic effects of endogenous and subcutaneous insulin. Under physiological conditions, insulin is secreted into the portal circulation and reaches the liver at high concentrations, where it suppresses gluconeogenesis and glycogenolysis while promoting glycolysis, glycogen synthesis and lipogenesis. By contrast, subcutaneously (s.c.) administered insulin enters the systemic circulation, resulting in altered tissue distribution, with lower and delayed hepatic exposure compared with endogenous insulin. See text for details. Created in BioRender.

Developing insulin derivatives and delivery strategies that more closely align with physiological patterns of insulin action therefore remains an important, albeit challenging, goal [26,27]. In this review, we discuss emerging approaches to engineer insulin molecules and delivery technologies that enable tissue- or site-specific insulin action, either to restore deficient signalling or to selectively enhance insulin effects where this may be therapeutically advantageous.

## 2. Insulin in the liver

As outlined above, the liver is the principal target organ for insulin [28]. The liver plays a central role in maintaining stable blood glucose levels, which is essential for the proper function—particularly of the brain, which rapidly deteriorates under conditions of insufficient glucose supply. In humans, fasting plasma glucose concentrations are normally maintained at approximately 4–6 mmol l^−1^ [29]. A decline below approximately 4 mmol l^−1^ (hypoglycaemia) is life-threatening, as insufficient glucose availability can rapidly and irreversibly impair brain function [30]. Conversely, chronic hyperglycaemia is also deleterious, as prolonged exposure to elevated glucose concentrations promotes non-enzymatic glycation of proteins, leading to progressive vascular and neural damage [31].

To maintain glucose homoeostasis, the liver maintains systemic glucose homoeostasis by rapidly releasing glucose from hepatic glycogen stores via glycogenolysis and by synthesizing glucose de novo through gluconeogenesis. At higher blood glucose concentrations, the liver stores glucose as glycogen [28], with both processes being tightly regulated by insulin. The liver also converts excess carbohydrates into fatty acids and triglyceride precursors (figure 2). Through these coordinated actions, insulin and hepatic metabolism restore elevated blood glucose levels towards the physiological range.

Unlike muscle and adipose tissue, which express the insulin-dependent glucose transporter GLUT4, hepatocytes primarily express GLUT2, a high-capacity, low-affinity transporter that is not regulated by insulin [32]. Consequently, glucose flux across the hepatocyte membrane is largely determined by the transmembrane glucose concentration gradient.

At the molecular level, insulin-mediated effects in the liver are primarily initiated by insulin binding to the insulin receptor (IR), leading to activation of its intrinsic tyrosine kinase activity and subsequent phosphorylation events that activate the insulin receptor substrate– (IRS)–PI3K–Akt signalling pathway [33,34]. In hepatocytes, IRS1 and IRS2 represent the principal mediators of insulin signalling, with IRS1 appearing to play a somewhat more prominent role in the regulation of hepatic glucose metabolism. Downstream of Akt, insulin signalling leads to phosphorylation and inactivation of FOXO transcription factors [35], which during fasting promote the expression of gluconeogenic genes. In parallel, insulin activates the transcription factor SREBP1c [36], thereby stimulating de novo fatty acid synthesis and lipogenesis (figure 3). Together, these mechanisms couple the suppression of hepatic glucose output to the storage of excess energy in the form of glycogen and lipids. Notably, IRS1/2 expression in the liver displays zonal heterogeneity, being enriched in pericentral hepatocytes [37], a distribution that may preferentially support glycolytic and lipogenic pathways in this region.

**Figure 3.**
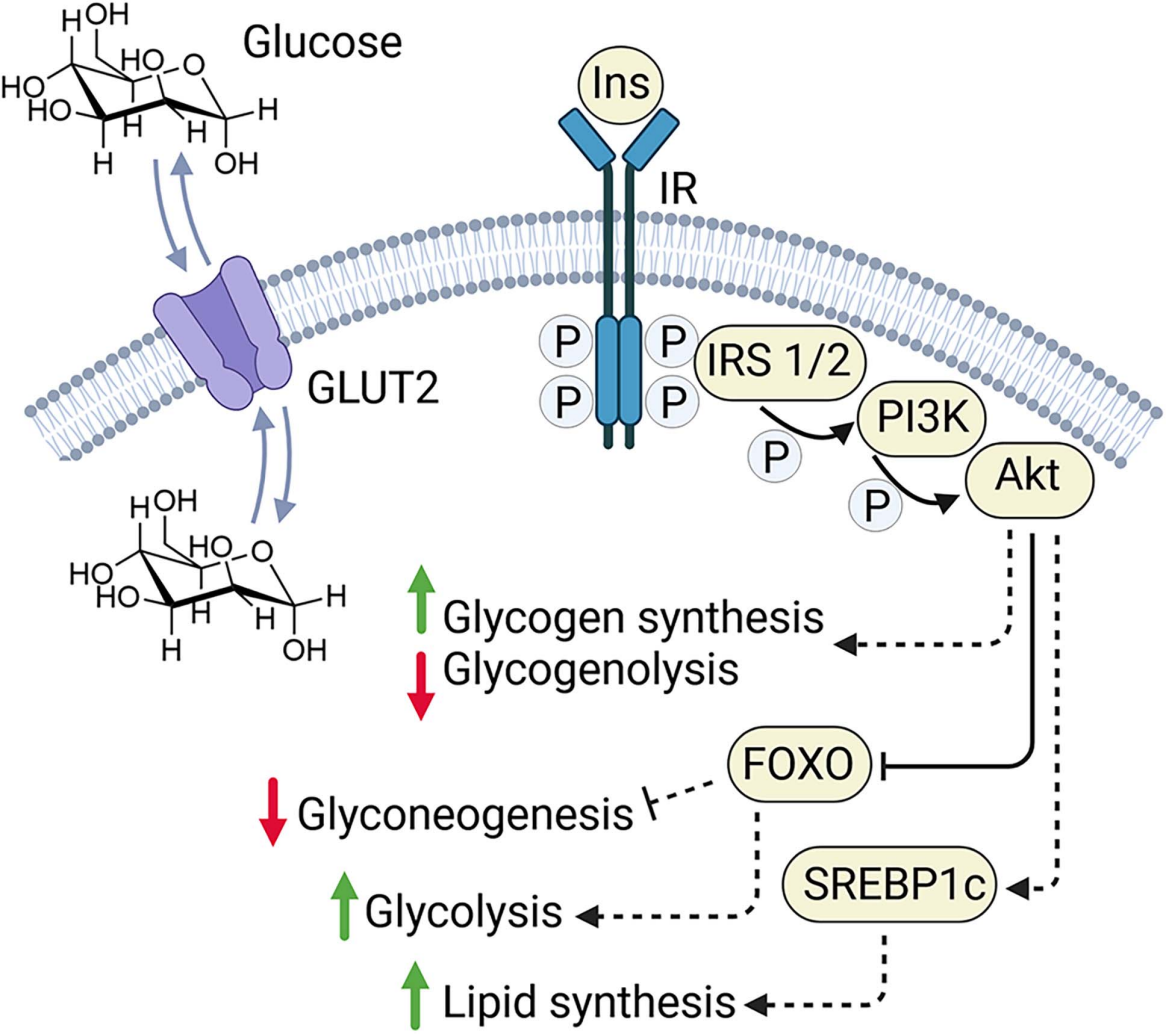
Simplified molecular mechanisms of insulin action in hepatocytes. Insulin (Ins) binding to its receptor (IR) activates the IRS–PI3K–Akt signalling pathway, with IRS1 and IRS2 acting as key mediators of downstream signalling. Activation of Akt leads to phosphorylation and inactivation of FOXO transcription factors, thereby suppressing gluconeogenic gene expression. In parallel, insulin stimulates the transcription factor SREBP1c, promoting *de novo* fatty acid synthesis and lipogenesis. Akt also promotes glycogen synthesis through multiple downstream mechanisms. These coordinated signalling events promote glycolysis and glycogen synthesis while suppressing gluconeogenesis and glycogenolysis, thereby coupling reduced hepatic glucose output to energy storage in the form of glycogen and lipids. Glucose transport across the hepatocyte membrane is mediated by GLUT2 and is largely insulin-independent, allowing bidirectional flux according to the glucose concentration gradient. Solid lines indicate direct signalling events, whereas dashed lines represent indirect or multistep regulatory mechanisms. Created in BioRender.

Importantly, insulin regulates hepatic metabolism through both direct and indirect mechanisms. Direct effects arise from insulin binding to hepatic IRs and subsequent activation of intracellular signalling cascades, as outlined in figure 3. Indirectly, insulin suppresses pancreatic glucagon secretion, inhibits adipose-tissue lipolysis—thereby limiting the availability of gluconeogenic substrates—and modulates hypothalamic insulin signalling that influences hepatic glucose production. In the absence of insulin and under low blood glucose conditions, glucagon acts as the principal driver of hepatic gluconeogenesis and glucose output. Consequently, insulin regulates hepatic metabolism through a combination of direct and, to a large extent, indirect pathways [28].

This regulatory architecture is physiologically important: when blood glucose levels are elevated, the liver should neither produce glucose nor mobilize it from glycogen stores. Accordingly, the preferential delivery of insulin from the pancreas to the liver via the portal vein is critically important. The liver extracts up to 50% of secreted insulin [38], while the remainder enters the systemic circulation, where it promotes glucose uptake in muscle and adipose tissue by stimulating GLUT4 translocation to the cell surface [39].

As mentioned above, therapeutic insulin is administered primarily by subcutaneous injection, where it precipitates and subsequently dissolves before entering the circulation. This route results in a relatively uniform insulin exposure across the liver, skeletal muscle and adipose tissue; however, hepatic insulin action is both reduced and delayed compared with the physiological portal delivery [27] (figure 2). Consequently, patients often experience suboptimal glycaemic control, which can contribute to long-term metabolic complications [40]. These limitations have driven substantial efforts to develop hepatoselective insulin analogues that, following subcutaneous administration, act more rapidly and physiologically in the liver, more closely mimicking physiological insulin distribution, improving fasting and postprandial glycaemia and reducing peripheral insulin exposure.

### 2.1. Hepato-preferential insulins through increased molecular size

In 1987, Lavelle-Jones and colleagues [41] demonstrated that human proinsulin exhibits a distinctly hepatoselective metabolic profile in dogs. Using anaesthetized greyhounds under a euglycemic clamp with somatostatin suppression of endogenous hormones, the authors showed that proinsulin reduced hepatic glucose output by approximately 50%, equivalent to insulin. However, unlike insulin, proinsulin failed to stimulate peripheral glucose use, which remained unchanged during proinsulin infusion, whereas insulin increased peripheral glucose disposal by roughly 50% above basal levels. These findings indicated that, at physiologically relevant concentrations, proinsulin acts predominantly through suppressing hepatic glucose production with minimal peripheral action.

Early attempts to create hepatoselective insulin derivatives were made by collaborating teams from Guy's and St Thomas's Hospitals in London and Deutsches Wollforschungsinstitut in Aachen [42], who tested whether increasing the molecular size of insulin analogues could shift their action preferentially towards the liver. They hypothesized that larger insulin species—such as the covalent dimers studied (*M_r_* ≈ 11 600) versus native insulin (*M_r_* ≈ 6000)—would show reduced access to peripheral tissues but preserved hepatic activity, consistent with earlier findings for proinsulin (*M_r_* ≈ 9000) [41]. In anaesthetized greyhounds under a euglycemic clamp, two dimers (linked at B1–B1′ or B29–B29′; figure 4) displayed markedly greater suppression of hepatic glucose production (*R_a_*) than stimulation of peripheral glucose disposal (*R_d_*). The B1–B1′ dimer retained 68% of insulin's potency for *R_a_* but only 14.7% for *R_d_*, and the B29–B29′ dimer showed 75% and 2.5%, respectively. Both also exhibited reduced metabolic clearance.

**Figure 4.**
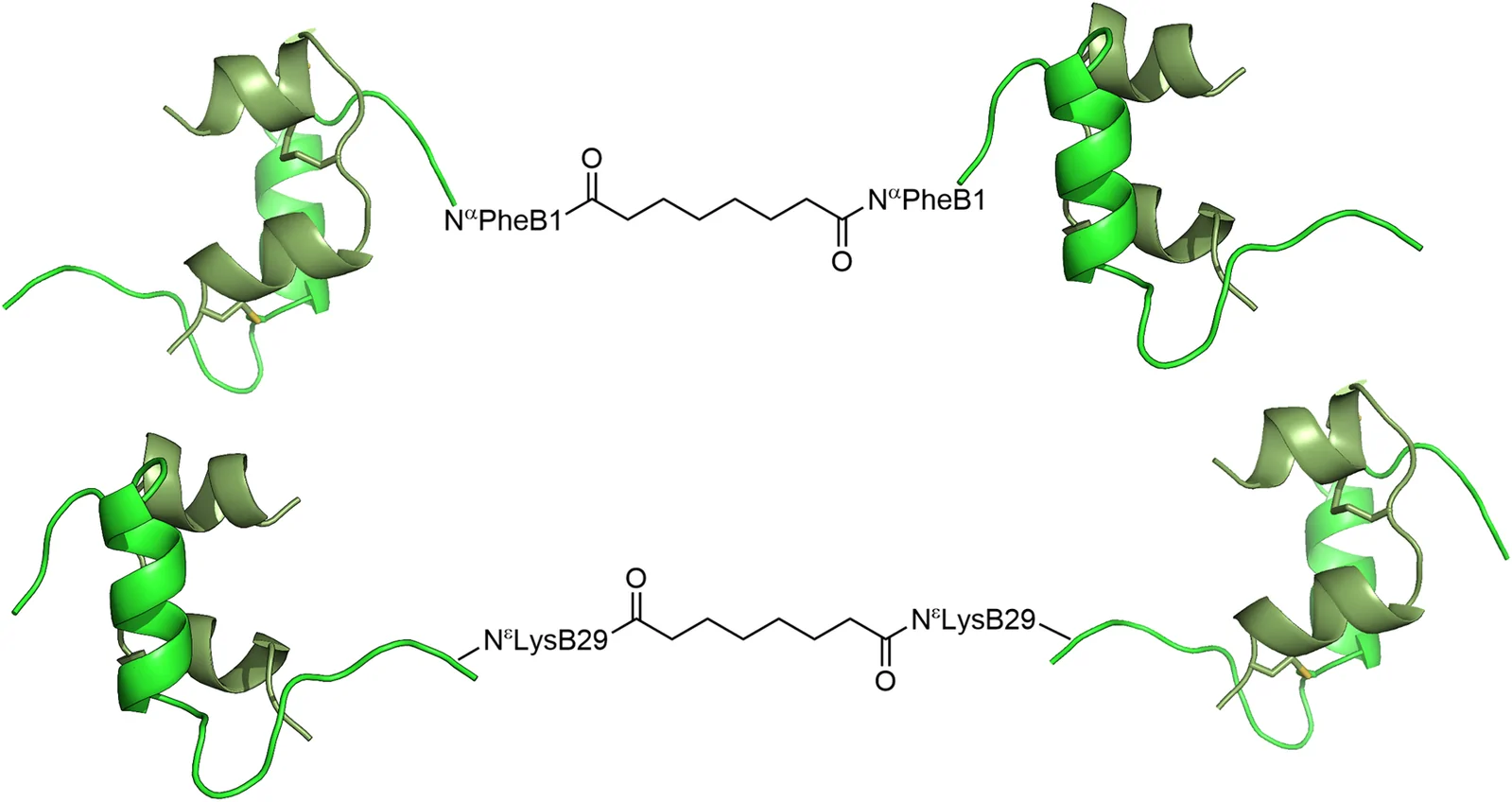
B1–B1′ (top), or B29–B29′ (bottom) suberoyl-linked insulin dimers [42]. The B-chain is in light green and A-chain in dark green; disulphides are not shown. Created in BioRender.

In 1998, the same teams tested whether insulin analogues that bind thyroid hormone-binding proteins (THBPs) could achieve relative hepatoselectivity [43]. They synthesized two conjugates of insulin with thyroxyl (figure 5) that formed high-molecular weight complexes with THBPs such as transthyretin, thyroxine-binding globulin and human serum albumin. In hyperinsulinemic euglycemic clamp studies in anaesthetized beagles, both analogues suppressed endogenous glucose production to an extent comparable to insulin, indicating preserved hepatic action, while their stimulation of peripheral glucose disposal was markedly reduced. These results supported the concept that THBP-bound insulin analogues retain preferential access to the liver while exhibiting limited peripheral activity, probably owing to restricted transcapillary transport. The teams also examined receptor binding of both intact conjugates and their THBP complexes using rat liver plasma membranes [44]. In the absence of THBPs, the conjugates bound with affinities similar to human insulin, whereas physiological concentrations of THBPs markedly impaired receptor binding. These findings indicate that only the dissociated (free) conjugates can engage the IR.

**Figure 5.**
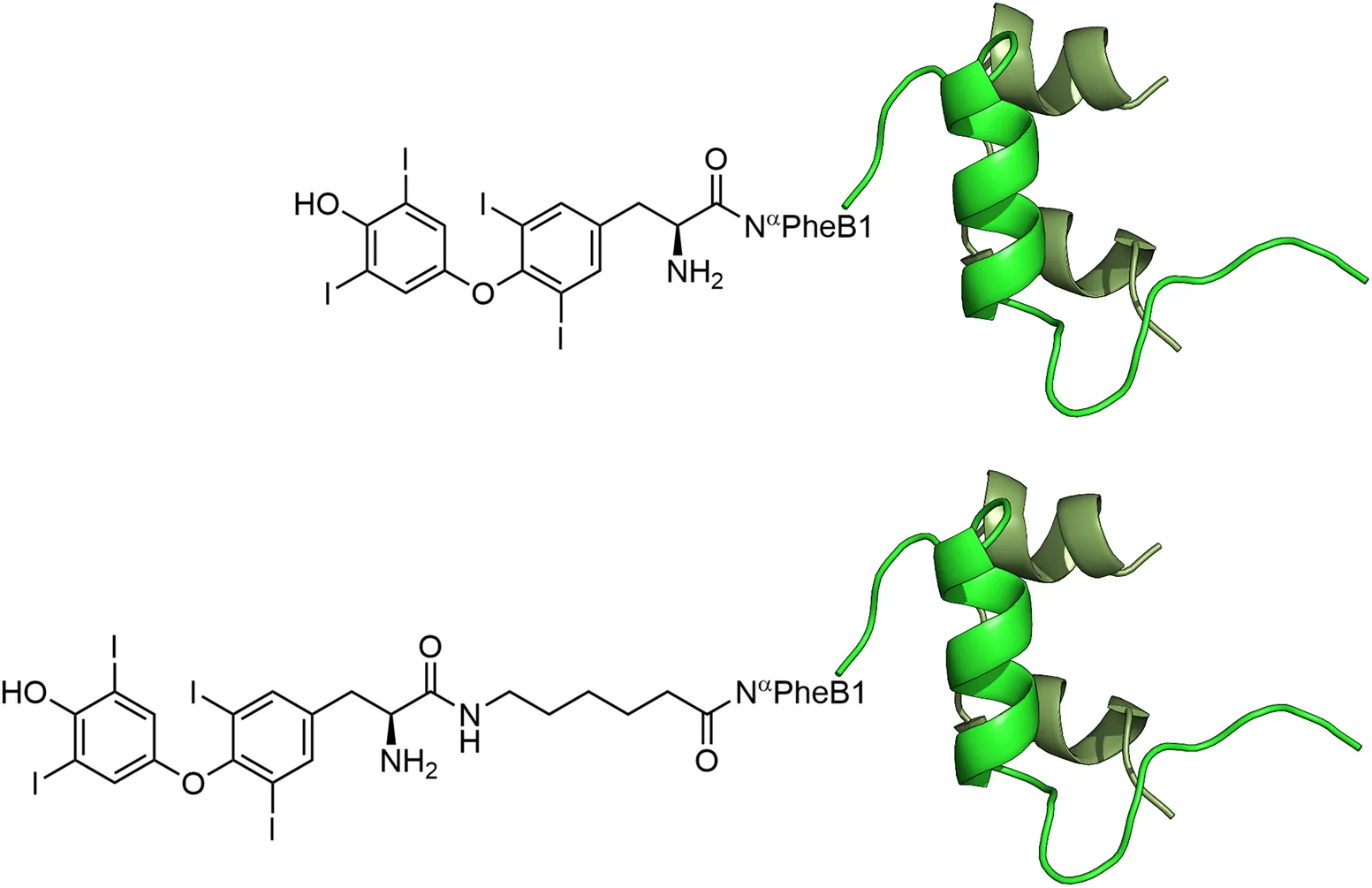
B1-thyroxyl insulin (top), and B1-aminohexanoic acid thyroxyl insulin (bottom) [43]. Created in BioRender.

Some degree of hepatoselectivity was also reported for insulin detemir (Levemir®, Novo Nordisk A/S), a long-acting insulin analogue modified with a myristic acid attached to the N^ε^ group of B29 lysine of *des*(ThrB30)-insulin (figure 6) [45].

**Figure 6.**
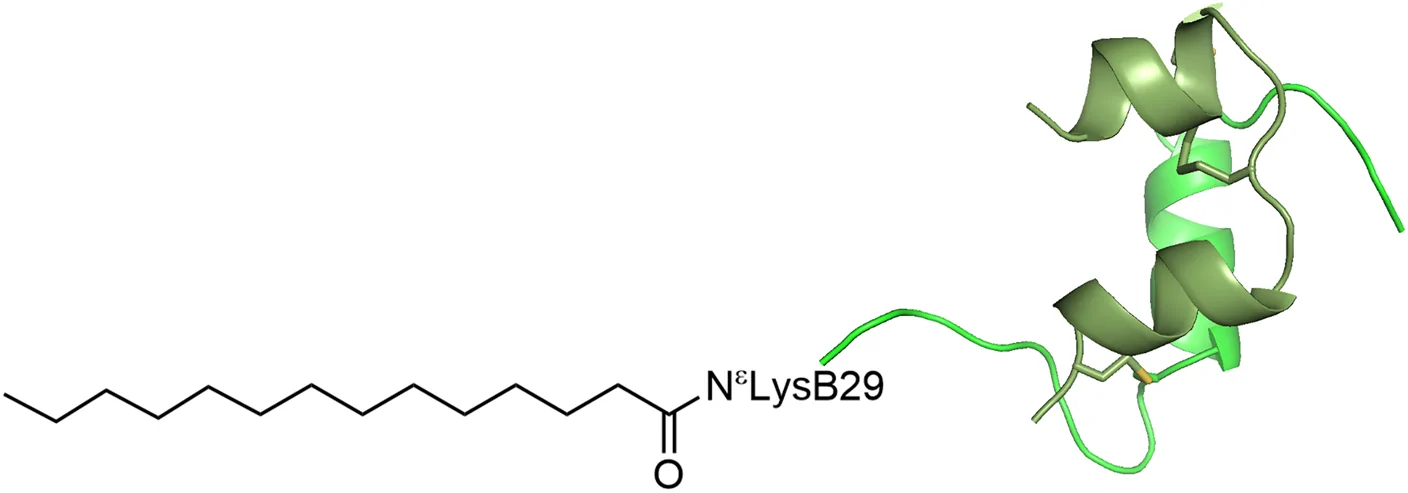
Insulin detemir [45]. Created in BioRender.

A proinsulin–transferrin fusion protein was also evaluated for its hepatopreferential activity in diabetic mice [46].

The tissue selectivity of the insulin derivatives described above is believed to result primarily from their increased molecular size, achieved either through molecular design (proinsulin and insulin dimers) or through binding to endogenous proteins (thyroxyl-insulin conjugates and insulin detemir). The fenestrated architecture of hepatic sinusoids facilitates the passage of macromolecules with an increased hydrodynamic radius into the liver, whereas their transport across the continuous capillary endothelium of skeletal muscle and adipose tissue is more restricted. However, hepatoselectivity is determined by several interrelated factors rather than molecular size alone. Plasma protein binding, IR affinity, receptor-mediated clearance and the overall pharmacokinetic properties of individual analogues also contribute to their tissue distribution and biological activity. Consequently, the hepatopreferential effects of some insulin derivatives probably reflect a combination of altered tissue distribution and substantially prolonged circulation time with reduced receptor-mediated clearance.

Building on the concept that albumin binding can direct a greater fraction of subcutaneously administered insulin to the liver, acylated insulin 406 was developed by Novo Nordisk A/S as a prandial hepato-preferential analogue with optimally balanced albumin binding and improved monomeric and metabolic stability. This analogue (figure 7) contains an engineered Lys residue at position A22 bearing an extended multifunctional linker composed of four *γ*-linked glutamate residues, a C14 diacid moiety, two ethylene glycol units, an additional *γ*-glutamate and a terminal C20 diacid and incorporates the substitutions GluB3, *des*(ThrB27), ArgB29 and *des*(ThrB30) [27]. In both preclinical models and individuals with type 1 diabetes, peripherally administered insulin 406 preferentially suppressed hepatic glucose production while exerting weaker effects on peripheral glucose uptake compared with conventional rapid-acting insulins [47]. Despite confirming a hepato-preferential metabolic profile, further clinical development of insulin 406 was discontinued for reasons that have not been publicly disclosed [27].

**Figure 7.**
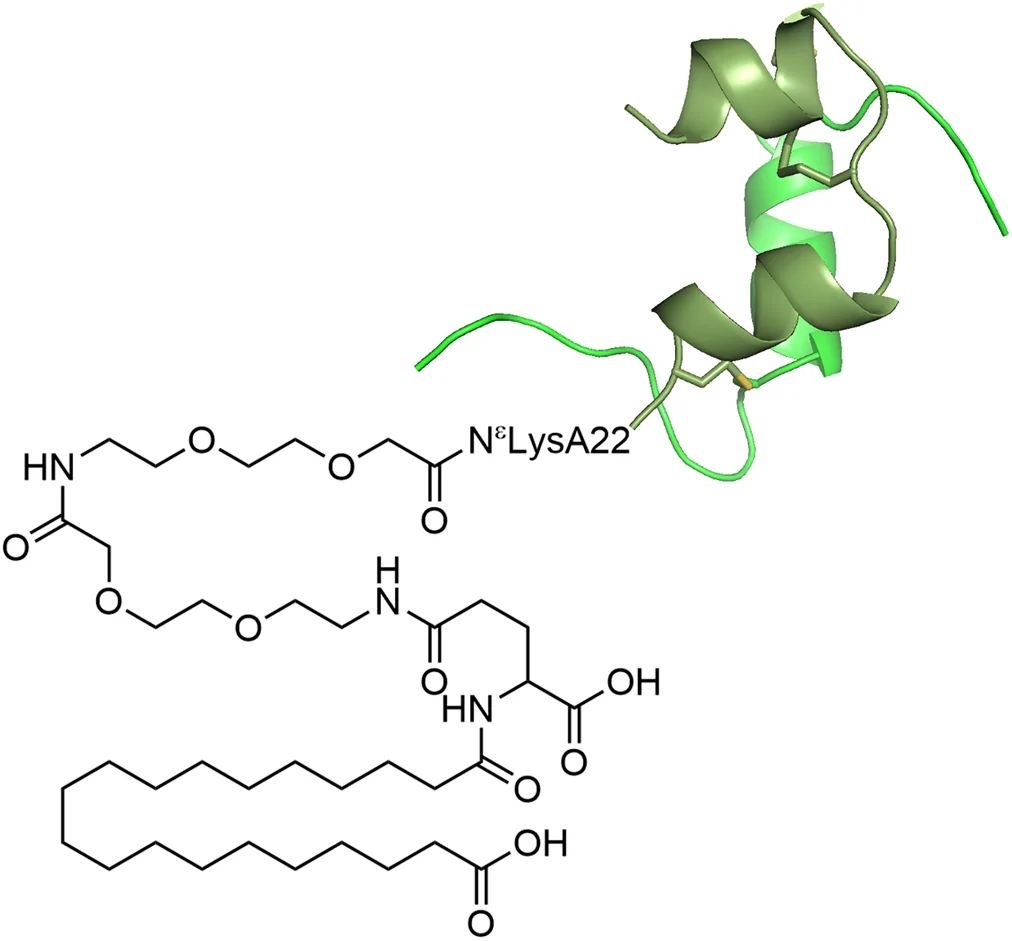
Insulin 406 [27,47]. Created in BioRender.

Eli Lilly developed insulin peglispro (BIL; LY2605541), a PEGylated derivative of insulin lispro (i.e. human insulin with a LysB28–ProB29 swap) created by attaching a 20 kDa linear polyethylene glycol chain to LysB28 (figure 8). While insulin lispro is a fast-acting analogue with reduced self-association [48,49], PEGylation markedly enlarges the molecule, resulting in prolonged circulation time owing to slower subcutaneous absorption and reduced renal clearance. Consistent with these properties, insulin peglispro displayed a long-lasting glucose-lowering effect in both preclinical models and clinical studies, with activity extending up to 36 h [50,51]. In patients with type 1 diabetes, it achieved satisfactory glycaemic control and showed a distinctive hepatoselective profile in euglycaemic clamp studies [52], characterized by a preferential suppression of hepatic glucose production. This more liver-focused action was associated with a lower incidence of nocturnal hypoglycaemia and modest weight reduction, an uncommon feature for insulin therapy [53]. However, concerns regarding elevated liver aminotransferase and triglyceride levels emerged during late-stage clinical development, ultimately leading Eli Lilly to discontinue the programme in 2014 [54].

**Figure 8.**
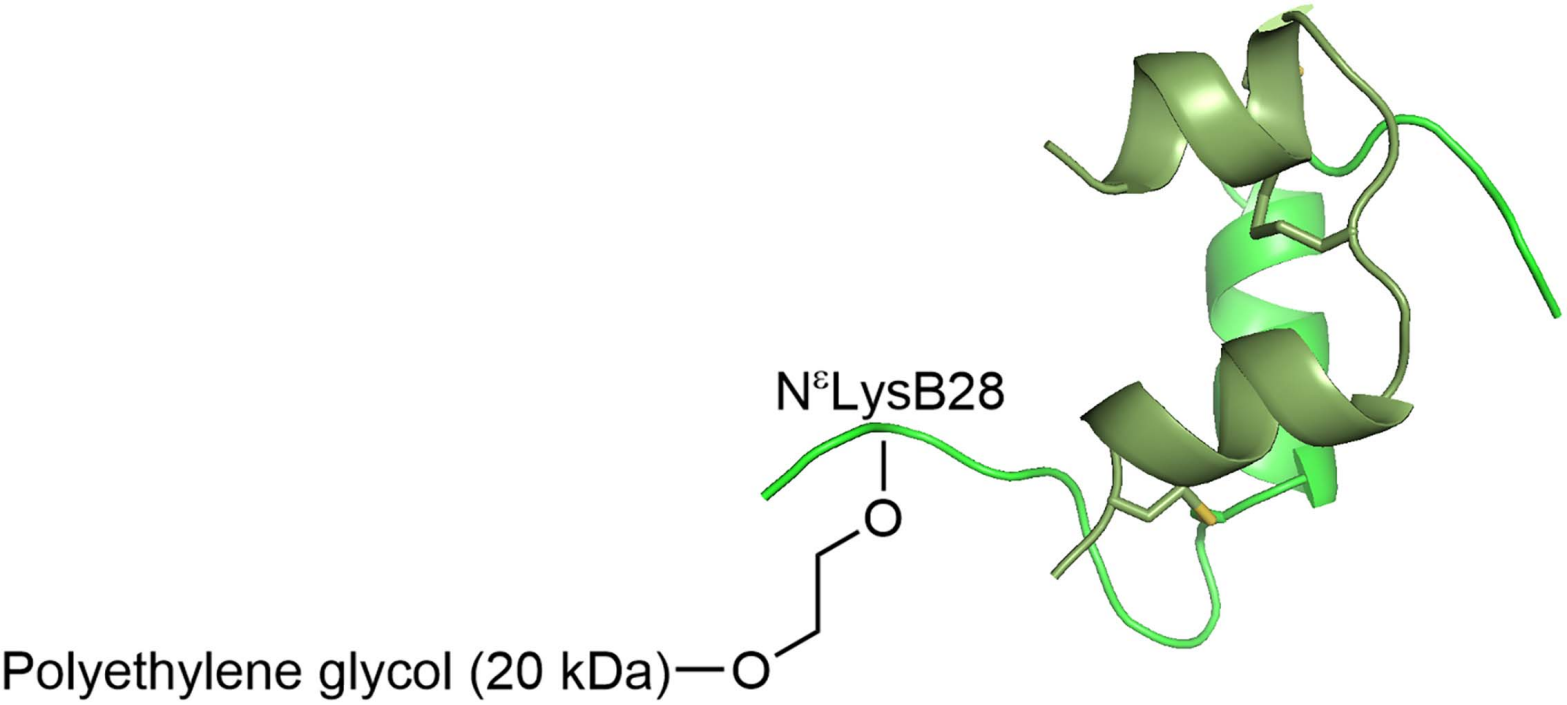
Structure of insulin peglispro (BIL; LY2605541), a PEGylated derivative of insulin lispro (i.e. human insulin with a LysB28–ProB29 inversion), generated by conjugation of a 20 kDa linear polyethylene glycol chain to N^ε^ of LysB28. Created in BioRender.

### 2.2. Hepato-preferential insulins through increased insulin receptor-B specificity

The IR is expressed as two isoforms generated by alternative splicing, which differ by the presence (IR-B) or absence (IR-A) of a 12-amino-acid segment at the C-terminus of the *α*-subunit (CT-peptide) [55,56]. Although small, this sequence is located close to the hormone-binding site and can therefore influence the binding of insulin, insulin-like growth factor 1 (IGF-1) and IGF-2 to the receptor (figure 9). Insulin exhibits slightly lower affinity for IR-B than for IR-A, whereas both IGFs bind to IR-B with markedly lower affinity than to IR-A [57]. The two isoforms also differ in the kinetics of insulin binding (*k*_on_ and *k*_off_) [58], which could affect downstream receptor signalling. Notably, the two isoforms display distinct tissue distributions: the liver expresses almost exclusively the IR-B isoform, whereas the brain and lymphoid tissues predominantly express IR-A, in both humans and rodents. Other tissues, such as skeletal muscle and adipose tissue, express varying proportions of IR-A and IR-B depending on the species [59,60]. It should be noted, however, that current knowledge of tissue-specific IR isoform expression is based largely on rodent studies, whereas information on their distribution in human tissues remains incomplete. This represents an important limitation and warrants systematic investigation of IR-A and IR-B expression across human tissues, including potential changes associated with ageing, metabolic status and disease.

**Figure 9.**
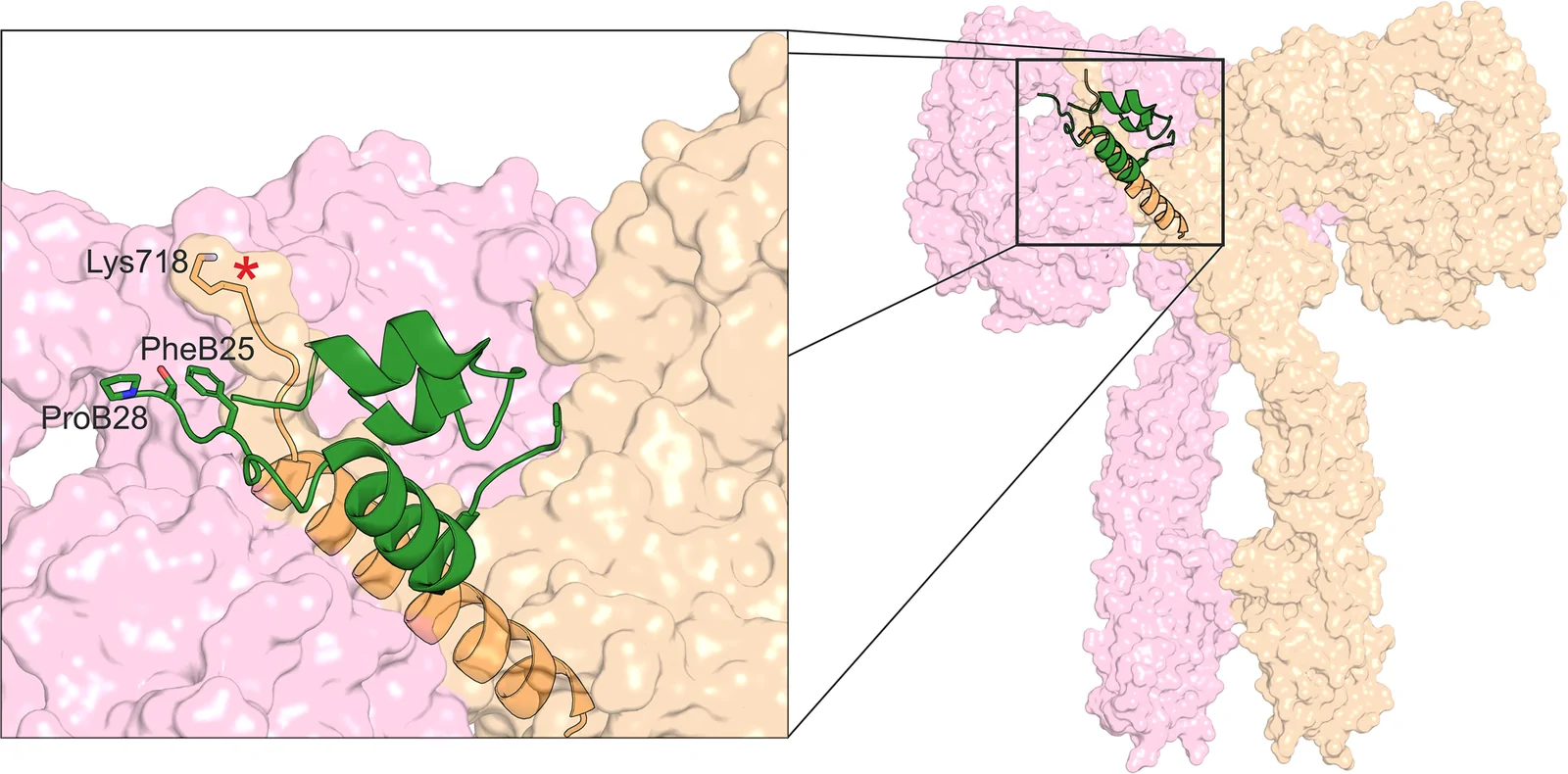
Simplified representation of the activated form of insulin receptor isoform B (IR-B), with the two protomers shown in beige and pink, bound to insulin (green). The inset shows a detail of insulin at binding site 1, where the hormone interacts closely with the C-terminal segment of the IR *α*-subunit (CT-peptide, coral). The red asterisk indicates the position of the insertion corresponding to residues 718–731 in human IR-B, which are not visible in the complex owing to their flexibility; these residues constitute the isoform-specific sequence absent in IR-A. Insulin B-chain residues PheB25 and ProB28 are also shown. Figure prepared in PyMOL based on structure 8U4C.

The physiological relevance of IR-A and IR-B remains a matter of debate and is not fully understood. However, owing to its nearly exclusive expression in the liver, IR-B is generally considered the receptor isoform primarily responsible for the metabolic effects of insulin, whereas IR-A is thought to be more closely associated with the mitogenic actions of insulin as well as those of IGF-1 and IGF-2, including in the context of cancer development [56]. Nevertheless, this distinction between the metabolic IR-B and the mitogenic IR-A isoform is an oversimplification. The two receptor isoforms share largely overlapping downstream signalling pathways, and the weaker signalling of IGF-1 and IGF-2 through IR-B mainly reflects their lower binding affinity for this isoform rather than fundamental differences in signalling properties [57].

The predominant presence of IR-B in the liver and the mixed population of both isoforms in peripheral tissues have led to the hypothesis that selectively modifying the binding affinities of insulin towards IR-A and IR-B in favour of the IR-B isoform could enhance hepatoselectivity *in vivo*. An insulin analogue with preserved or increased affinity for IR-B and, ideally, reduced affinity for IR-A might be less efficiently captured by IR-A after subcutaneous administration, thereby allowing a greater fraction of the hormone to reach the liver. This could translate into more favourable glycaemic outcomes compared with human insulin or conventional fast-acting or long-acting insulin analogues. In addition, reduced affinity for IR-A may also reduce the potential risk of excessive mitogenic effects during chronic insulin therapy.

Based on these considerations, a Novo Nordisk A/S team engineered two insulin analogues, INS-A and INS-B, with altered binding specificities towards IR isoforms [60,61]. INS-A (B(1–29)-Val-Gly-Leu-Ser-Ser-Gly-Gln-A(1–21)-GlnA18 human insulin, that is *des*(ThrB30) human insulin with a connecting Gly-Leu-Ser-Ser-Gly-Gln linker connecting LysB29 to GlyB1 and Asn-to-GlnA18 mutation), exhibits approximately 55% of the binding potency of human insulin for IR-A but only about 10% for IR-B. Its single-chain structure, resembling that of proinsulin or IGFs, confers weak binding to IR-B by introducing steric hindrance with the longer CT-peptide of IR-B. By contrast, INS-B (HisA8, AsnB25, GluB27 and *des*(ThrB30) human insulin) displays roughly 9% of the binding affinity of human insulin for IR-A and about 28% for IR-B, making it approximately threefold selective for IR-B while retaining low affinity for IR-A. Its IR-B selectivity was engineered by modifying amino acids predicted to interact with regions of the CT-peptides that differ between IR-B and IR-A.

Using a combination of *in vitro* and *in vivo* approaches, Vienberg *et al.* [60] demonstrated that isoform-selective receptor binding translates into differential metabolic effects depending on tissue-specific IR expression. In primary rat hepatocytes and adipocytes, which predominantly express IR-B, INS-B showed higher potency than human insulin in stimulating glycogen accumulation and lipogenesis, whereas INS-A was less effective. Conversely, in rat skeletal muscle, where IR-A is the dominant isoform, INS-A exhibited relatively greater potency for glycogen synthesis compared with its effects in liver and fat. Acute *in vivo* studies in mice further supported tissue-selective signalling. When administered intravenously at doses producing comparable glucose-lowering effects, INS-B induced significantly greater IR phosphorylation in the liver than human insulin, whereas no major differences were observed in skeletal muscle. These findings indicate that IR-isoform-preferential insulin analogues can bias insulin action towards specific tissues *in vivo*. The study also highlights that IR isoform expression outside the liver and brain is not conserved across species, which may limit direct translation to humans. Overall, the work of Vienberg *et al.* [60] provided proof of principle that modifying insulin receptor interactions can generate tissue-preferential metabolic responses and offers a conceptual framework for developing insulin therapies that more closely mimic physiological insulin distribution.

In 2021, Páníková *et al.* [62] revitalized the concept of IR-B–specific insulin analogues. They designed and tested a large series of insulin analogues modified at the C-terminus of the B-chain of the hormone. Within this series, several insulin analogues with markedly increased binding specificity towards IR-B were identified. The insulin analogue AlaB29-GluB31-B31-carboxamide exhibited approximately 40% affinity for IR-A and 140% affinity for IR-B relative to human insulin, making it more than threefold selective for IR-B while retaining high affinity for this isoform and reduced affinity for IR-A. This analogue also displayed increased resistance to thermal denaturation compared with human insulin. It may therefore represent a promising candidate for further investigation of its effects under *in vivo* conditions.

### 2.3. Concluding remarks on perspectives of hepatopreferential insulins

To date, considerable scientific effort has been devoted to the development of insulin derivatives with increased hepatoselectivity, with the aim of improving glycaemic control in patients with diabetes. Available evidence indicates that a hepatoselective effect can, in principle, be achieved even after peripheral administration, either by increasing the molecular size of insulin or by modulating its selectivity towards IR isoforms.

Indeed, several studies have shown that insulin analogues with an increased IR-B-to-IR-A binding affinity ratio and reduced affinity for IR-A relative to human insulin exhibit promising *in vitro* and preclinical profiles, consistent with enhanced hepatoselectivity [60,62]. In particular, hepato-preferential insulin analogues such as insulin peglispro [52,53] or insulin 406 [27,47], which have been evaluated in clinical studies, demonstrated favourable pharmacodynamic properties and appeared to more closely mimic physiological insulin action. These analogues offered potential advantages, including improved glycaemic control, reduced weight gain and a lower risk of hypoglycaemia.

Nevertheless, in the case of promising insulin peglispro, which progressed to phase III clinical trials, safety concerns ultimately limited its further development. These concerns included persistently elevated liver aminotransferase levels, increased hepatic fat content as assessed by magnetic resonance imaging and higher circulating triglyceride concentrations compared with insulin glargine. Despite favourable glycaemic outcomes, these hepatic and lipid-related liabilities led to discontinuation of its clinical development in 2016 [54].

In addition, excessive hepatoselectivity may compromise insulin action in skeletal muscle and adipose tissue. Consistent with clamp studies showing reduced stimulation of peripheral glucose disposal despite preserved suppression of endogenous glucose production, such an imbalance could impair postprandial glucose handling and may therefore require co-administration of a prandial, peripherally acting insulin component [63]. From a conceptual standpoint, an optimal strategy may thus involve a dual approach: a mildly hepatopreferential basal insulin combined with a conventional prandial insulin.

Taken together, these findings highlight important considerations for the clinical development and evaluation of hepato-preferential insulin analogues, particularly with regard to defining their overall benefit–risk profile. Importantly, these limitations do not argue against the continued development of hepatopreferential insulin derivatives *per se* but rather underscore the need for careful fine-tuning of their pharmacological properties.

## 3. Insulin in the brain

The brain is highly dependent on a continuous supply of glucose, which is ensured by tight regulation of blood glucose levels within a narrow physiological range, primarily through the coordinated actions of insulin and the liver (see above). Glucose entry into the brain occurs predominantly via facilitated diffusion across the BBB mediated by GLUT1, followed by neuronal uptake through the high-affinity transporter GLUT3 [32]. Because cerebral glucose uptake is largely insulin-independent, the brain was historically considered an insulin-insensitive organ.

However, growing evidence suggests that insulin plays numerous important roles in the CNS. Although it has long been assumed that insulin present in the adult mammalian brain originates exclusively from the circulation [64,65], recent studies suggest that insulin may also be synthesized within specialized neuronal populations and neural circuits of the CNS [66,67]. The IR is expressed in many regions of the adult brain [64]. Most IRs in the brain are located in neurons, which express almost exclusively the IR-A isoform, whereas glial cells express lower levels of IR-A and predominantly the IR-B isoform [68,69]. The presence of IR in human brain endothelial cells suggests that insulin may cross the BBB, at least in part, via receptor-mediated transcytosis.

It is now well established that the brain is an insulin-sensitive organ in which the hormone elicits multifaceted actions and regulates behavioural and metabolic responses to internal and external cues through coordinated interactions between peripheral and central insulin signalling pathways. Insulin influences proliferation, survival and differentiation of multiple brain cell types. Although insulin does not directly regulate neuronal glucose uptake, it modulates hypothalamic control of whole-body carbohydrate and lipid metabolism, cellular fuel handling, tissue remodelling, feeding behaviour and body weight. In addition, insulin modulates synaptic plasticity, synapse density, neurotransmission and the formation of neural circuits governing food intake, energy allocation, reproductive behaviour, and cognition, learning, and memory across the lifespan. Collectively, these actions link central insulin signalling tightly to brain energy homoeostasis [64,70–74]. The multifaceted roles of insulin in the brain, encompassing its transport across the BBB, cell type–specific receptor expression and functional effects on neuronal activity and systemic energy homoeostasis, are summarized schematically in figure 10.

**Figure 10.**
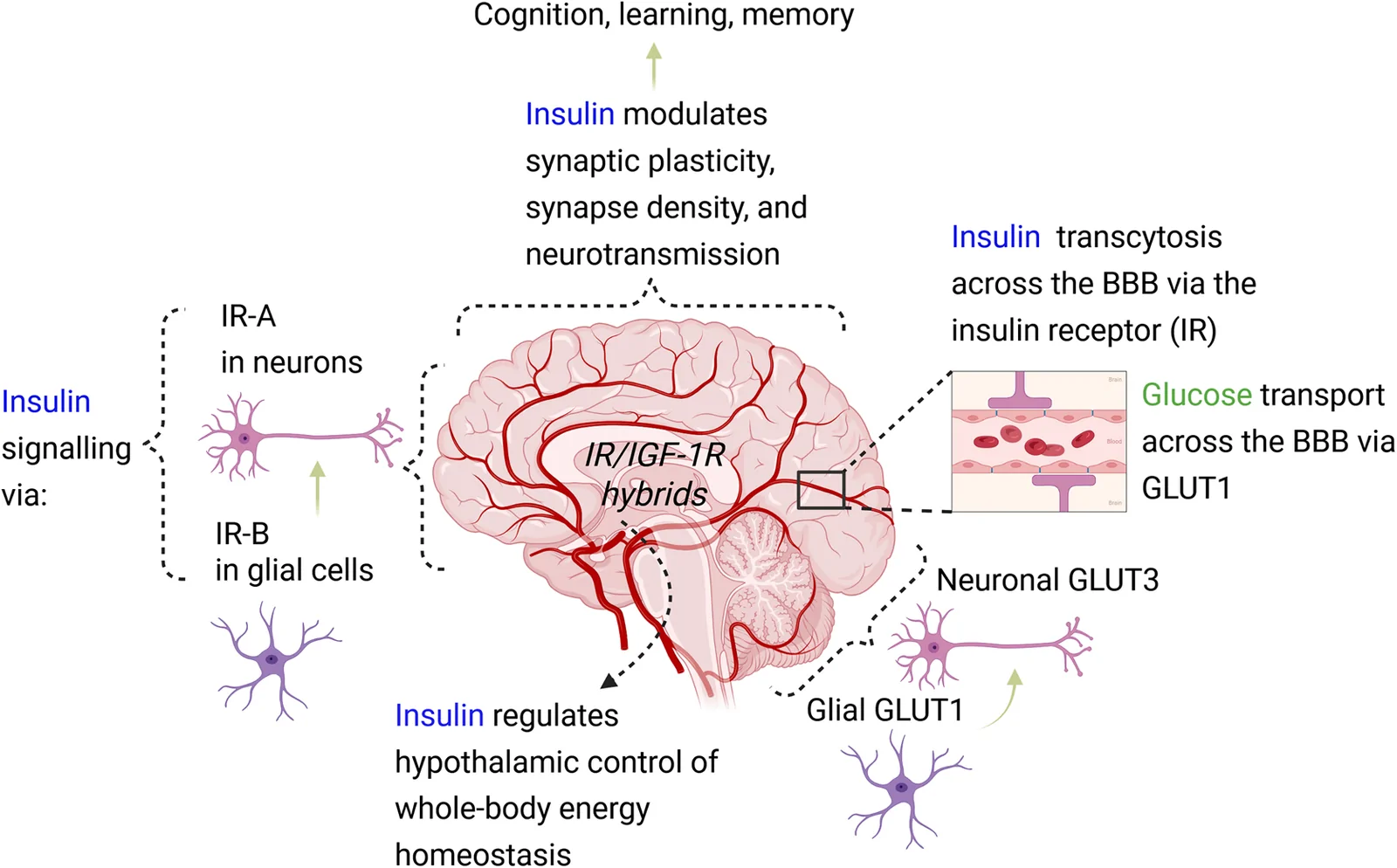
Insulin action and glucose transport in the brain. Glucose entry into the brain occurs primarily via facilitated diffusion across the blood–brain barrier (BBB) mediated by GLUT1, followed by uptake into neurons through the high-affinity transporter GLUT3, while glial cells predominantly express GLUT1. In contrast to glucose transport, which is largely insulin-independent, insulin reaches the brain in part via insulin receptor-mediated transcytosis across the BBB. Within the central nervous system, insulin action is mediated by IR-A, which is predominantly expressed in neurons, and IR-B, which is enriched in glial cells that provide metabolic and structural support to neurons. Insulin modulates synaptic plasticity, synapse density and neurotransmission, thereby contributing to higher brain functions such as cognition, learning and memory. In addition, insulin regulates hypothalamic control of whole-body energy homoeostasis, linking central and peripheral metabolic processes. Co-expression of IR and insulin-like growth factor 1 receptor (IGF-1R) may give rise to hybrid receptors in the brain; however, their physiological significance remains incompletely understood [75–78]. Created in BioRender.

The generation of a brain-specific IR knock-out mouse model in 2000 [79] demonstrated that loss of neuronal insulin signalling results in hyperphagia, obesity and the development of whole-body insulin resistance [70]. These findings gave rise to the concept of brain insulin resistance, extending classical models of type 2 diabetes mellitus (T2DM) to a bidirectional interaction between central and peripheral insulin action. In this framework, insulin resistance in peripheral tissues may impair insulin signalling in the brain and *vice versa*, creating a self-reinforcing pathological cycle [74]. Targeting brain insulin resistance has therefore been proposed as a potential strategy for the prevention and treatment of metabolic diseases such as T2DM.

Substantial epidemiological and experimental evidence links insulin resistance in T2DM with an increased risk of Alzheimer's disease (AD), and multiple studies indicate impaired central insulin signalling in AD patients [80,81]. Nevertheless, it remains unresolved whether a T2DM-like state in the brain is a cause or a consequence of AD pathology. Insulin dysfunction may predispose to AD, whereas AD-related pathology may, in turn, predispose to diabetes [82]. Disturbances in brain insulin action therefore represent a plausible mechanistic link between metabolic and cognitive health. Beyond AD, impaired insulin signalling has also been described in other neurodegenerative disorders, including Huntington's and Parkinson's diseases [64]. Overall, improving central insulin action may represent a therapeutic opportunity for individuals at increased risk of metabolic and cognitive disorders. In line with this concept, treatment of experimental models of neurodegenerative diseases with insulin has shown neuroprotective effects and functional benefits [83,84].

It is now well established that the brain is not only sensitive to insulin, but that the related growth factors IGF-1 and IGF-2 also play important roles in the CNS [64,70–74,85]. Both insulin and IGFs influence the proliferation, survival and differentiation of multiple brain cell types. Dysregulation of insulin signalling has been reported not only in AD but also in other neurodegenerative disorders, including Huntington's and Parkinson's diseases [64]. Consequently, improving central insulin and IGF signalling may represent a promising therapeutic strategy for individuals at increased risk of metabolic and cognitive disorders [81]. In line with this, treatment of experimental models of neurodegeneration with insulin [83], IGF-1 [86,87] or IGF-2 [88,89] has produced beneficial outcomes and demonstrated neuroprotective effects of these hormones.

A somewhat overlooked aspect of insulin and IGF physiology is that co-expression of the IR and IGF-1 receptor (IGF-1R) results in the formation of so-called hybrid receptors, composed of one IR protomer and one IGF-1R protomer. These hybrids are thought to form according to Siddle's random assembly model, depending on the relative expression levels of each receptor within the cell [90]. Because hybrid receptors bind insulin with considerably lower affinity than IR homodimers but retain high affinity for IGF-1 [75,78], their presence can influence the relative responsiveness of tissues to insulin and IGFs. As a consequence, tissues expressing a high proportion of hybrid receptors may respond relatively less to insulin and more to IGF-1 than tissues dominated by IR homodimers.

Hybrid receptors are not restricted to the CNS but have been identified in numerous insulin-responsive tissues. However, they are particularly abundant in several brain regions where both IR and IGF-1R are co-expressed. Increasing evidence suggests that coordinated activation of IR, IGF-1R and hybrid receptors contributes to the regulation of brain metabolism, neuronal survival and central control of glucose homoeostasis and may also be relevant to neural insulin resistance during ageing and neurodegenerative disease [76,77]. These observations suggest that the ability to activate hybrid receptors may represent an attractive property of future insulin analogues designed for tissues expressing high levels of IR/IGF-1R hybrids.

In this context, a recently developed insulin analogue ([GluB10, D-HisB24, GlyB31, TyrB32]-insulin; **1**_Ins_) may have particularly interesting applications. This analogue is equipotent with IGF-1 in binding to IGF-1R and shows higher affinity for both IR-A and IR-B than human insulin. It activates these receptors in cells more efficiently than the native hormones and exhibits neuroprotective effects in neuronal cell models. A recent study demonstrated that at least part of these effects is mediated by hybrid receptors (figure 11) [91].

**Figure 11.**
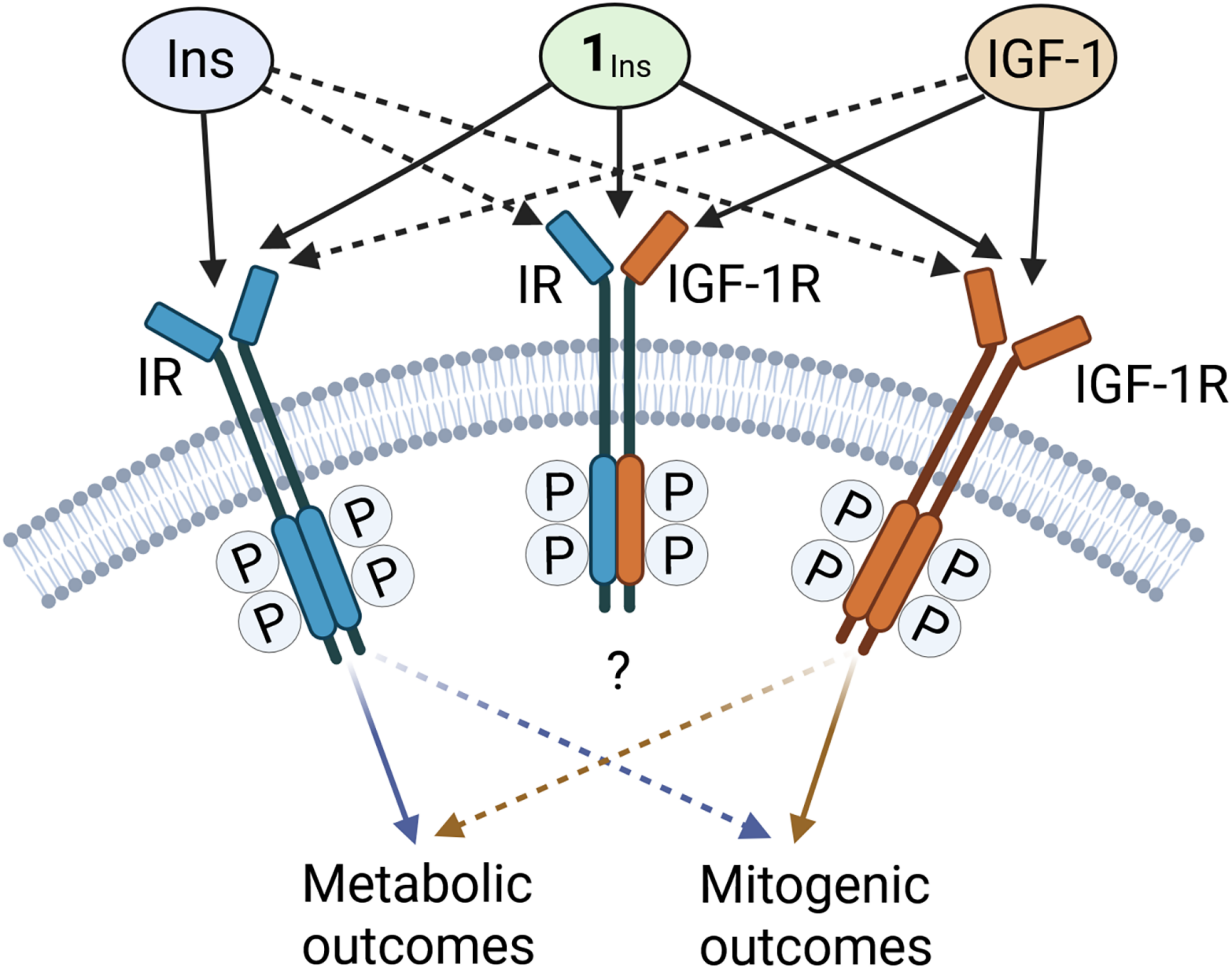
Differential activation of insulin receptor (IR), IGF-1 receptor (IGF-1R) and hybrid IR/IGF-1R receptors by insulin, IGF-1 and the insulin analogue 1_Ins_. The insulin receptor (IR, shown in blue) and IGF-1 receptor (IGF-1R, shown in orange) can form homodimeric receptors or hybrid receptors composed of one IR and one IGF-1R protomer. Ligand binding is indicated by arrows, with solid lines representing preferential interactions and dashed lines indicating lower-affinity binding. Insulin primarily activates IR, while IGF-1 preferentially activates IGF-1R. The insulin analogue **1**_Ins_ ([GluB10, D-HisB24, GlyB31, TyrB32]-insulin) binds and activates both receptors with high affinity and is capable of engaging hybrid receptors [91]. IR signalling is more strongly associated with metabolic functions, whereas IGF-1R signalling is predominantly associated with growth and mitogenic responses. By contrast, the signalling outputs of hybrid receptors remain incompletely understood, as indicated by the question mark. Created in BioRender.

### 3.1. Selective targeting of insulin to the brain

Insulin has a very narrow therapeutic index [26], meaning that there is only a small margin between a dose that produces the desired physiological effect and a dose that may cause harm. This is primarily owing to its high efficacy in lowering blood glucose levels, where excessive dosing can lead to hypoglycaemia and subsequent brain damage. This characteristic complicates the use of peripherally administered insulin for targeted applications in the CNS. Ideally, approaches that enable selective delivery of insulin to the brain while minimizing peripheral exposure would therefore be highly desirable.

Intranasal delivery of insulin, first introduced in the early 2000s [92], has been a key enabling technology for probing insulin's direct actions in the human CNS while largely avoiding the major limitation of peripheral insulin administration—namely the risk of systemic hypoglycaemia owing to insulin's narrow therapeutic window in peripheral tissues. In contrast to peripheral dosing, intranasal insulin exploits so-called nose-to-brain transport routes, primarily along olfactory and trigeminal pathways, providing relatively rapid CNS exposure while largely avoiding significant changes in circulating insulin and glucose [93].

This approach has therefore been widely adopted as an experimental and translational tool to study how insulin modulates brain functions related to memory and cognition, energy balance and obesity, and potentially brain insulin resistance and its links to neurodegeneration. A landmark placebo-controlled pilot trial in adults with amnestic mild cognitive impairment or mild-to-moderate AD demonstrated that several months of intranasal insulin administration could produce measurable cognitive and functional signals. In this study (*n* = 104), daily intranasal insulin administered for four months at doses of 20 IU or 40 IU (1 IU corresponds to 6 nmol or approximately 34 µg of insulin) improved delayed memory at the 20 IU dose and preserved carer-rated functional ability at both doses, with exploratory biomarker associations suggesting links to AD-relevant cerebrospinal fluid measures. Importantly, fluorodeoxyglucose-positron emission tomography (FDG-PET) imaging in a subset of participants revealed an insulin-associated attenuation of the decline in cerebral glucose metabolism in regions typically affected in AD, supporting the notion that augmenting CNS insulin signalling may influence regional brain glucose metabolism in humans [94].

A critical inflection point for the field came from a large multi-site phase 2/3 randomized clinical trial (*n* = 289 participants across 27 sites; 12-month blinded treatment plus extension). In the primary analyses, intranasal insulin did not demonstrate cognitive or functional benefit compared with placebo over 12 months. Notably, however, the authors explicitly reported that study execution and interpretation were complicated by technical problems with the intranasal delivery device. Among the first 49 participants, the initial device exhibited inconsistent reliability, prompting a switch to a second device that was subsequently used for the remaining 240 participants comprising the primary intention-to-treat population. These device-related limitations complicated the interpretation of the trial and underscored the need for delivery systems that can reliably and demonstrably elevate insulin levels in the CNS [95].

More recently, molecular imaging studies have provided direct evidence that intranasally administered insulin reaches key memory- and cognition-related regions of the human brain. In a first-in-human PET study, radiolabelled insulin delivered via a specialized nasal delivery system was detected in multiple regions associated with memory, including the hippocampus, olfactory cortex, amygdala and temporal lobe, in older adults. Importantly, systemic blood glucose levels remained stable, demonstrating that intranasal delivery largely targeted insulin to the CNS without inducing peripheral hypoglycaemia [96]. This work provided direct molecular imaging evidence of insulin delivery to the human brain, rather than relying solely on functional or cognitive readouts.

Although intranasal delivery currently represents the most advanced approach for selective CNS targeting of insulin, systemic BBB-targeting strategies are also beginning to emerge. An alternative strategy for selectively targeting insulin to the brain could involve its conjugation or molecular coupling to a transport-enabling protein capable of mediating rapid passage across the BBB following systemic administration. Such approaches aim to exploit receptor-mediated transcytosis pathways expressed on brain endothelial cells, thereby enhancing CNS exposure while minimizing peripheral effects. A prominent proof of principle for this concept was recently demonstrated in the context of AD therapy [97], where an anti–amyloid-*β* monoclonal antibody was engineered into an antibody transport vehicle by incorporating transferrin receptor (TfR) binding directly into the immunoglobulin G Fc fragment domain. This TfR-targeted architecture enabled efficient capillary-mediated transcytosis across the BBB, resulting in broad parenchymal brain distribution and substantially improved target engagement compared with a conventional antibody. Importantly, this delivery route shifted biodistribution away from large cerebral arteries and reduced vascular adverse effects, illustrating how molecular engineering of biologics to engage endogenous BBB transport systems can profoundly alter brain exposure and safety profiles.

### 3.2. Concluding remarks on insulin targeting to the brain

Taken together, accumulating evidence supports the view of insulin as a multifunctional hormone in the brain that integrates metabolic, cognitive and behavioural processes. While intranasal delivery has been instrumental in elucidating central insulin actions in humans, its clinical translation has been constrained by variability in delivery efficiency and device performance, highlighting the need for further well-controlled clinical studies. Emerging molecular engineering strategies that enable selective transport of biologics across the BBB offer a complementary and conceptually attractive avenue for targeting insulin to the CNS. Future development of insulin analogues or insulin-based conjugates optimized for brain delivery may therefore provide new opportunities to better understand central insulin signalling with greater precision and to explore its therapeutic potential in metabolic and neurodegenerative disorders, while minimizing peripheral adverse effects.

## 4. Insulin in skeletal muscle

Skeletal muscle is among the most metabolically dynamic tissues and represents approximately 40–50% of total body mass. Functionally, it is the dominant site of whole-body insulin-stimulated glucose disposal and therefore a key determinant of postprandial glycaemic control. In response to feeding-associated hyperinsulinaemia, skeletal muscle rapidly increases glucose uptake and channels glucose into glycolysis for ATP production or into glycogen synthesis for storage, thereby buffering circulating glucose excursions. These integrated actions are orchestrated by IR signalling pathways that converge on GLUT4 vesicle trafficking and on enzymes controlling carbohydrate metabolism and glycogen storage [98].

At the cellular level (figure 12), insulin promotes GLUT4 translocation to the plasma membrane primarily through PI3K–Akt signalling and downstream regulation of Rab GTPase modules that control vesicle mobilization, targeting and fusion. In muscle cells, Rab8a (together with Rab13) has been implicated as an insulin-activated regulator of GLUT4 trafficking downstream of AS160/TBC1D4, consistent with a role in discrete steps of GLUT4 vesicle transport and tethering near the plasma membrane [99]. Beyond glucose handling, insulin also exerts major anabolic effects in muscle by increasing amino acid uptake and stimulating net protein synthesis, in part via nutrient delivery through microvascular recruitment and activation of mTORC1-dependent signalling [100].

**Figure 12.**
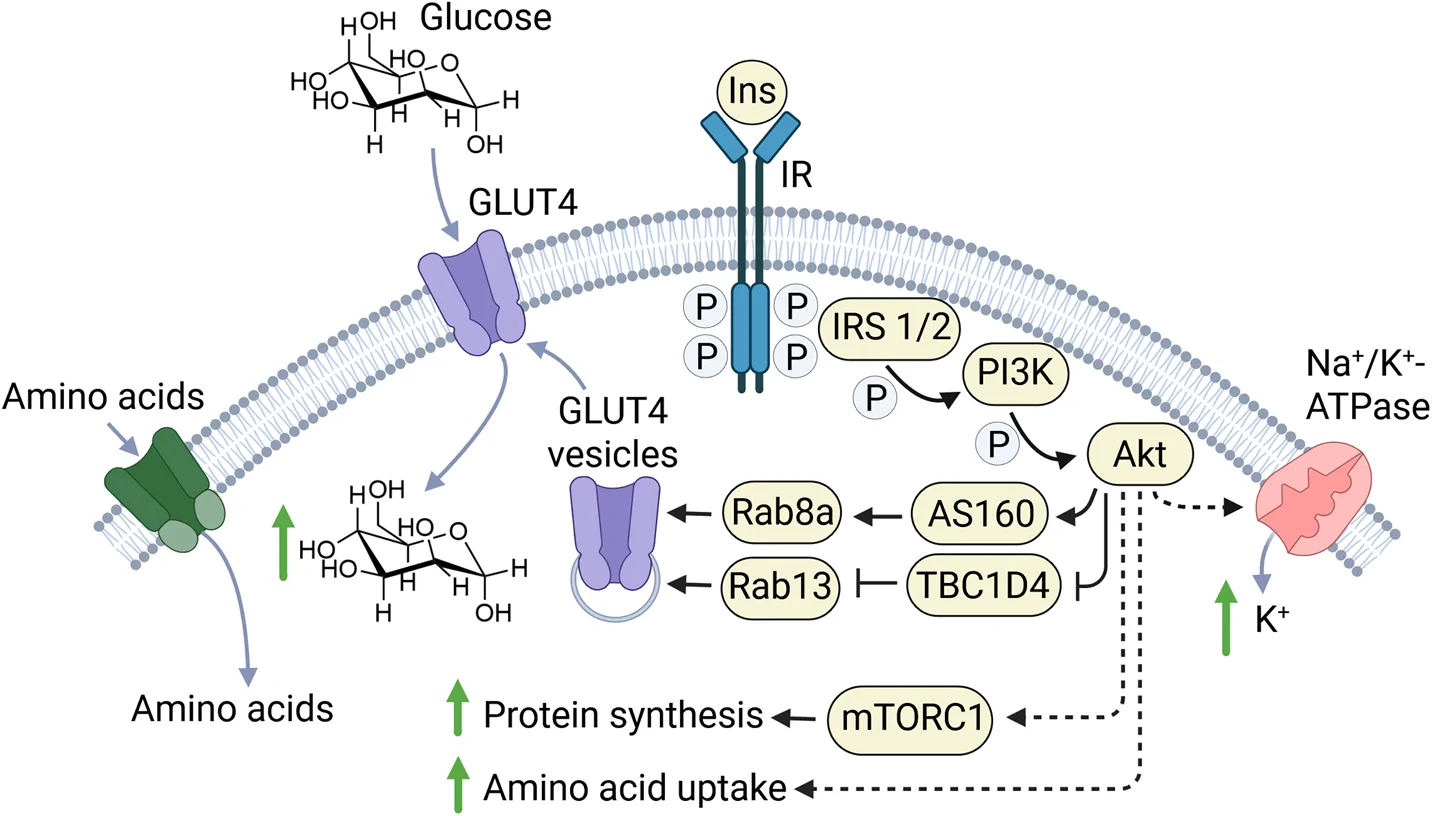
Simplified insulin signalling and metabolic regulation in a muscle cell. Insulin (Ins) binding to the insulin receptor (IR) activates the IRS1/2–PI3K–Akt signalling cascade. Akt promotes GLUT4 translocation to the plasma membrane through phosphorylation of AS160/TBC1D4, relieving its inhibitory effect on Rab GTPases (including Rab8a and Rab13), which regulate GLUT4 vesicle trafficking, targeting and fusion. This leads to increased glucose uptake. In parallel, insulin stimulates cellular K⁺ uptake in part via regulation of Na⁺/K⁺-ATPase activity. Insulin also promotes anabolic processes by activating mTORC1-dependent signalling, leading to increased protein synthesis. Insulin also enhances amino acid uptake through indirect mechanisms, thereby increasing intracellular amino acid availability and contributing to mTORC1 activation. Black solid lines indicate direct interactions or transport processes, whereas black dashed lines represent indirect or multistep regulatory mechanisms. Created in BioRender.

Insulin signalling in skeletal muscle depends strongly on adaptor proteins of the IRS family. Genetic disruption studies indicate that combined loss of IRS1 and IRS2 in striated muscle produces profound defects in muscle growth and metabolic programming, associated with impaired Akt–mTOR signalling and altered AMP-activated protein kinase activity, underscoring the essential role of IRS-dependent insulin/IGF signalling in maintaining muscle mass and metabolic homoeostasis [101]. In parallel, insulin action in muscle is closely linked to lipid and amino acid metabolism (including circulating branched-chain amino acids), and it contributes to electrolyte homoeostasis by stimulating cellular K⁺ uptake—classically attributed to increased Na⁺/K⁺-ATPase activity and membrane recruitment [102]—mechanisms that are clinically relevant to insulin-induced hypokalaemia.

### 4.1. Muscle-selective insulin: rationale, opportunities and caveats

Conceptually, a muscle-selective insulin (or an insulin analogue with preferential activity in skeletal muscle) could enhance peripheral glucose disposal and thereby improve postprandial control at lower systemic exposure. This might reduce non-muscle collateral insulin actions that contribute to weight gain, fluid retention or excessive suppression of lipolysis outside muscle, while potentially supporting muscle metabolic fitness (e.g. glycogen storage and insulin responsiveness) with chronic dosing.

However, increasing muscle bias also raises predictable physiological risks. If hepatic suppression of endogenous glucose production is insufficient, fasting glycaemia could worsen, implying that any strongly muscle-selective strategy may need to be combined with at least a modest hepatic component. In addition, because muscle glucose uptake is highly sensitive to perfusion and contraction state, exercise could amplify its metabolic effects in an activity-dependent manner and increase hypoglycaemia variability. Finally, given that insulin drives K⁺ into muscle, a more muscle-skewed pharmacology could theoretically accentuate hypokalaemia risk in predisposed individuals (e.g. those with arrhythmia vulnerability or receiving potassium-lowering therapies). A further system-level concern is metabolic ‘mismatch’: weaker inhibition of adipose lipolysis could raise circulating free fatty acids (FFAs), which may secondarily impair hepatic insulin sensitivity and counteract glycaemic benefit [103,104]. These limitations could, in principle, be mitigated by combination approaches, for example co-administration of a hepatoselective insulin analogue to ensure adequate suppression of hepatic glucose production while preserving the benefits of enhanced muscle glucose disposal.

### 4.2. Approaches explored towards muscle-directed insulin action

Unlike hepatoselective insulin analogues, which have already entered clinical development, muscle-targeted insulin remains largely conceptual, and no clinically validated targeting strategy is currently available. Nevertheless, several conceptually distinct approaches have been proposed to shift insulin action towards skeletal muscle. One strategy is to engineer ligands that bias downstream pathway activation towards metabolic signalling (e.g. Akt-linked pathways) relative to mitogenic signalling (e.g. mitogen-activated protein kinase (MAPK)), resulting in functionally selective (‘biased’) insulins [105]. Although functional bias towards Akt relative to MAPK signalling does not confer tissue selectivity *per se*, it may preferentially support acute metabolic responses in skeletal muscle, where insulin-stimulated glucose uptake is highly dependent on Akt-mediated GLUT4 translocation [106]. By contrast, mitogenic and other long-term transcriptional insulin actions rely more strongly on MAPK signalling [107] and may therefore be relatively attenuated.

In this context, Novo Nordisk's basal insulin analogue NNC-965 (NNC0268-0965; insulin 965), described as carrying four amino acid substitutions and a C18 fatty diacid—although detailed structural information is not publicly available—was reported in a conference abstract [108] to exhibit a higher Akt than MAPK activation profile in several cellular systems and *in vivo*. Early clinical evaluation included multiple phase 1 trials (e.g. first-in-human NCT03965013, T2D PK/PD NCT04136067 and a 26-week randomized study versus insulin glargine NCT04575181). However, no detailed results from these studies have been publicly reported to date, and further clinical development has not been disclosed.

Targeted delivery strategies represent an alternative approach to enhance the action of biologics in skeletal muscle by exploiting tissue-specific ligands, antibodies or depot-based formulations. In this context, transferrin receptor 1 (TfR1/CD71) has been explored as an entry point for muscle-directed delivery [109]. Although most work in this area has focused on oligonucleotide therapeutics, proof-of-concept studies have demonstrated that TfR1-mediated targeting can enable efficient delivery to skeletal muscle and cardiac tissue and, depending on ligand properties and affinity, has also been used for receptor-mediated transport across the BBB using specific anti-TfR1 antibodies (see above; [97]).

In parallel, short muscle-homing peptides have been identified through *in vivo* and phage-display screening approaches. A commonly cited example is the heptapeptide ASSLNIA, which was originally selected for its ability to enhance adeno-associated virus serotype 2 tropism towards striated muscle [110]. In addition, CAR (CARSKNKDC) is a wound-homing peptide targeting angiogenic neovessels in regenerating tissues and has been used as a delivery vehicle to injured sites; recent work further links CAR activity to syndecan-4–dependent trafficking and signalling pathways implicated in tissue repair [111], supporting its potential use for targeting cargos (e.g. anabolic and growth-promoting insulin-like peptides) to regenerating rather than healthy muscle. Targeting of skeletal muscle via cell-surface receptors has also been explored using peptide motifs with affinity for muscle-expressed proteins such as *α*-dystroglycan, where A2G80-modified carriers show enhanced accumulation in dystrophic muscle after systemic administration [112,113].

### 4.3. Concluding remarks on insulin targeting to skeletal muscle

In summary, skeletal muscle represents an attractive but inherently complex target for insulin modulation. While hepatoselective insulin analogues aim to restore physiological control of endogenous glucose production, muscle-directed strategies focus on enhancing peripheral glucose disposal, particularly in the postprandial state. Beyond metabolism, insulin also supports muscle anabolism and regeneration, suggesting that locally enhanced insulin action may influence muscle repair under specific conditions. Although functional signalling bias alone is unlikely to confer true tissue selectivity, emerging receptor-guided delivery platforms and muscle-homing ligands may enable preferential delivery of insulin or insulin-like peptides to skeletal muscle. The clinical use of such approaches will depend on their ability to complement hepatic insulin action while preserving metabolic safety.

## 5. Insulin in adipose tissue

Adipose tissue is a major metabolic organ responsible for storing excess energy in the form of triglycerides and releasing energy substrates when required. In addition to this storage function, adipose tissue acts as an endocrine organ that secretes numerous adipokines, including leptin, adiponectin and inflammatory mediators, which participate in the regulation of systemic metabolism and insulin sensitivity. Through these combined metabolic and endocrine roles, adipose tissue contributes importantly to the maintenance of whole-body energy homoeostasis and is closely linked to the development of metabolic disorders such as obesity and T2DM [114].

Insulin is the principal hormonal regulator of adipocyte metabolism [115] (figure 13). Insulin exerts its effects in adipose tissue through both IR isoforms. IR-A is predominantly expressed in mesenchymal and less-differentiated cells and is more closely associated with proliferative and developmental processes, whereas IR-B is more abundant in terminally differentiated cells and is primarily linked to metabolic signalling [116]. Following nutrient intake and the associated rise in circulating insulin levels, insulin stimulates glucose uptake into adipocytes mainly by inducing translocation of the insulin-dependent glucose transporter GLUT4 to the plasma membrane. The increased intracellular glucose supply provides glycerol-3-phosphate required for triglyceride synthesis. At the same time, insulin promotes fatty-acid uptake and stimulates lipogenesis, thereby facilitating energy storage in adipose tissue [115].

**Figure 13.**
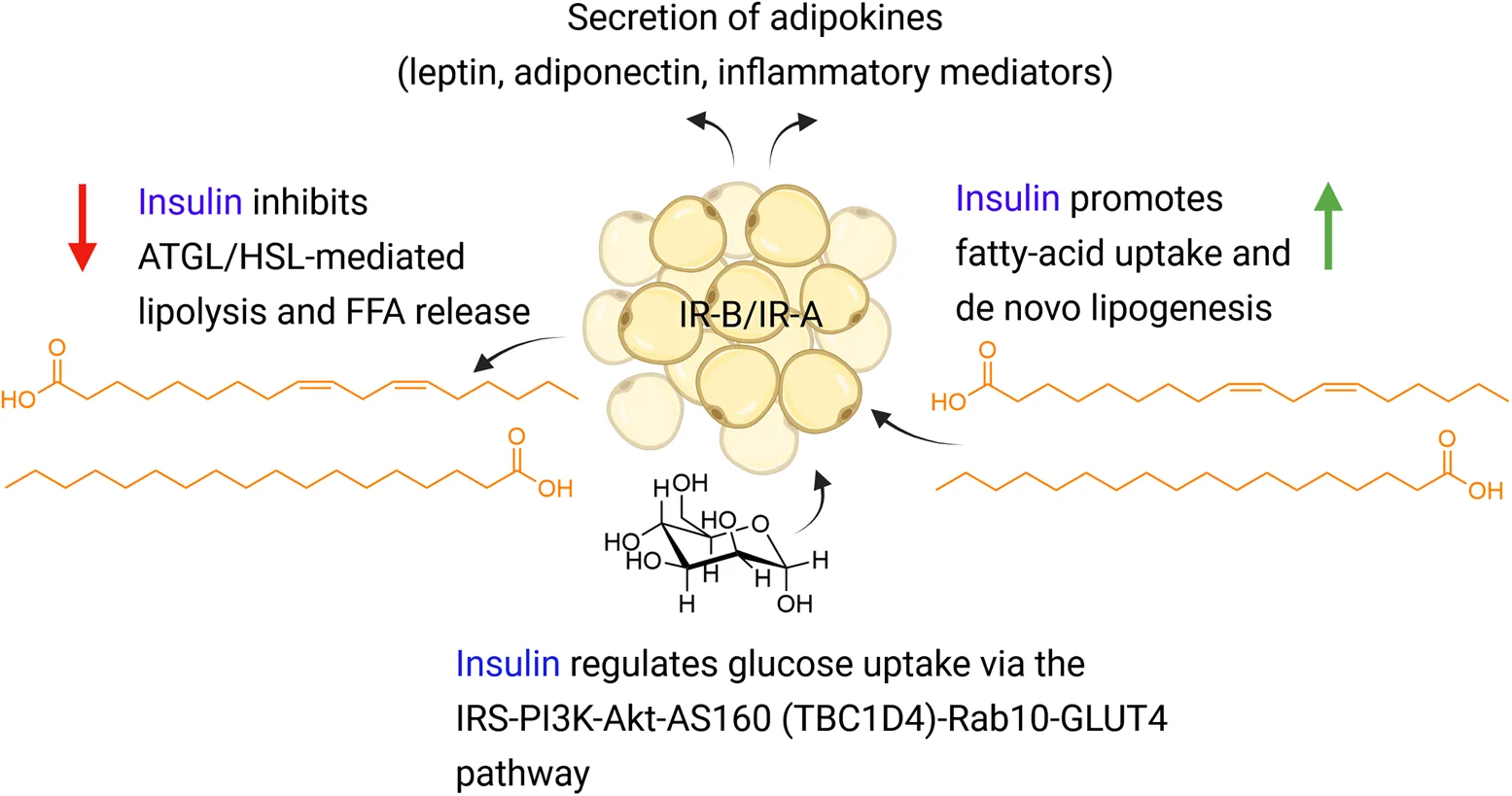
Insulin action in adipocytes. Adipose tissue functions as both a metabolic and endocrine organ, storing excess energy in the form of triglycerides and secreting adipokines, including leptin, adiponectin and inflammatory mediators. Insulin exerts its effects through the insulin receptor isoforms IR-A and IR-B, with IR-B predominantly mediating metabolic signalling in differentiated adipocytes. Activation of the IRS–PI3K–Akt pathway promotes GLUT4 translocation to the plasma membrane via AS160 (TBC1D4) and Rab10 GTPase, thereby enhancing glucose uptake. The resulting increase in intracellular glucose provides glycerol-3-phosphate required for triglyceride synthesis. In parallel, insulin stimulates free fatty acid (FFA) uptake and de novo lipogenesis while suppressing ATGL/HSL-mediated lipolysis, thereby limiting the release of FFAs. Created in BioRender.

Equally important is the inhibitory effect of insulin on lipolysis. In the fasting state, triglycerides stored in adipocytes are hydrolysed by lipases such as adipose triglyceride lipase (ATGL) and hormone-sensitive lipase (HSL), releasing FFAs and glycerol into the circulation. Insulin suppresses this process by inhibiting lipolytic activity and thereby limiting the release of FFAs. This regulation has important systemic consequences, as increased FFA flux from adipose tissue promotes hepatic gluconeogenesis, lipid accumulation in liver and skeletal muscle, and the development of insulin resistance [117].

At the molecular level, insulin signalling in adipocytes [118] is mediated primarily through activation of the IR and the downstream IRS–PI3K–Akt pathway. Activation of Akt results in phosphorylation of AS160/TBC1D4 and subsequent activation of Rab GTPases involved in GLUT4 vesicle trafficking, including RAB10. Through these mechanisms, insulin rapidly enhances glucose transport into adipocytes. In parallel, insulin signalling regulates enzymes controlling lipid synthesis and suppresses lipolytic pathways, thereby coordinating glucose uptake with triglyceride storage.

Adipose tissue therefore plays a key systemic role by regulating circulating FFA concentrations. Elevated plasma FFAs impair insulin signalling in liver and skeletal muscle and are considered an important contributor to whole-body insulin resistance [118,119].

### 5.1. Adipose tissue-selective insulin: rationale, opportunities, caveats and conclusions

From the perspective of tissue-selective insulin action, modulation of insulin signalling in adipose tissue could influence metabolic control indirectly through changes in lipid flux. Certain pathological states may therefore benefit from enhanced insulin action in adipose tissue. In conditions such as lipodystrophy or adipose insulin resistance, impaired lipid storage capacity leads to excessive release of FFAs and ectopic lipid accumulation in liver and skeletal muscle. In such settings, restoring insulin sensitivity in adipose tissue may improve systemic metabolic homoeostasis by promoting lipid storage and suppressing lipolysis. However, excessive stimulation of adipose insulin signalling may also promote lipid accumulation and expansion of adipose tissue.

At present, strategies for adipose tissue-selective insulin delivery remain largely hypothetical, and the concepts discussed below should therefore be regarded primarily as future opportunities. Selective delivery of insulin to adipose tissue could, in principle, be achieved using adipose-homing ligands or carrier-based targeting strategies. The best characterized examples include prohibitin-binding peptides that target white adipose tissue vasculature [120], as well as adipocyte-binding aptamers [121] or nanoparticle-based delivery systems [122]. However, to our knowledge, these approaches have not yet been explored for adipose-targeted insulin delivery, and their application to insulin remains largely conceptual.

Conversely, attenuation of insulin action in adipose tissue may also be of potential interest, particularly in the context of obesity. It may be speculated that hepato-preferential insulin analogues could indirectly reduce insulin action in adipose tissue, thereby limiting lipid storage and weight gain. However, because insulin signalling in adipose tissue is also required to suppress lipolysis and restrain systemic FFA flux, excessive reduction of adipose insulin action may compromise overall metabolic homoeostasis. In support of this concept, fat-specific IR knock-out mice display reduced adipose mass and are protected against age-related obesity [123]. However, more severe models of adipose IR deficiency develop lipodystrophy, hyperlipidaemia and fatty liver, illustrating that excessive suppression of adipose insulin signalling can become metabolically detrimental [124].

Any therapeutic strategy aimed at selectively modulating insulin action in adipose tissue would therefore require careful balance of these opposing metabolic effects.

## 6. Systemic consequences of tissue-selective insulin action

Although the individual tissue-targeting strategies have been discussed separately throughout this review, insulin physiology should be viewed as an integrated endocrine system rather than a collection of independent tissue-specific effects. Consequently, selective modulation of insulin action in one tissue inevitably influences the function of other tissues and should therefore be considered in the context of whole-body metabolic regulation [115,125]. Several examples illustrate this inter-organ crosstalk. Excessive hepatoselectivity may improve suppression of hepatic glucose production but at the same time reduce the contribution of skeletal muscle to postprandial glucose disposal. Likewise, altered insulin action in adipose tissue affects lipolysis and the release of FFAs, thereby secondarily modifying insulin sensitivity in both the liver and skeletal muscle. Central insulin signalling also contributes to the regulation of hepatic glucose production through brain–liver communication. Conversely, excessive stimulation of glucose uptake by skeletal muscle without appropriate suppression of hepatic glucose production may increase the risk of hypoglycaemia, while enhanced insulin-stimulated potassium uptake into muscle may predispose to hypokalaemia. These examples illustrate that the metabolic consequences of tissue-selective insulin analogues extend beyond the primary target organ. Evaluation of novel tissue-targeting strategies should therefore consider not only the desired effects within the target tissue but also their impact on inter-organ metabolic crosstalk and whole-body metabolic homoeostasis.

## 7. Conclusion

Tissue-selective modulation of insulin action represents a promising strategy to improve the therapeutic profile of insulin beyond conventional systemic delivery. As illustrated across the liver, brain, skeletal muscle and adipose tissue, insulin exerts highly context-dependent effects that are determined by tissue-specific receptor expression, intracellular signalling networks and physiological function. In addition to the major metabolic target tissues discussed in this review, insulin also exerts important actions in vascular endothelial and immune cells. Although these topics were beyond the scope of the present review, they represent important and rapidly evolving areas of insulin research and may provide additional opportunities for future tissue-targeting strategies. Efforts to bias insulin action towards the liver and the brain—whether through molecular engineering of insulin analogues, modulation of receptor isoform specificity or targeted delivery approaches—have demonstrated proof of principle in both preclinical and clinical settings. By contrast, tissue-selective targeting of skeletal muscle and adipose tissue remains largely at the conceptual stage, highlighting important opportunities for future research. However, the complexity of whole-body metabolic regulation imposes important constraints. Excessive targeting towards a single tissue may disrupt the coordinated balance between hepatic glucose production, peripheral glucose disposal and lipid metabolism, potentially leading to unintended metabolic consequences. Future strategies will probably require finely tuned approaches that preserve systemic homoeostasis while enhancing tissue-preferential effects. In this context, continued advances in protein engineering, delivery technologies and a systems-level understanding of insulin action may enable the development of next-generation insulin therapies that more closely recapitulate physiological insulin distribution and function.

## Data Availability

This article has no additional data.
